# Nutritional Status, Frailty, Oral Health, and Oral Motor Functions in Long-Term Care Residents with Swallowing Dysfunction

**DOI:** 10.3390/jcm14010062

**Published:** 2024-12-26

**Authors:** Chih-Hung Ko, Chia-Ling Chao, Chih-Hsing Hung, Je-Kang Du, Ming-Chu Feng

**Affiliations:** 1 Department of Psychiatry, Kaohsiung Municipal Siaogang Hospital, Kaohsiung Medical University, Kaohsiung 812, Taiwan; 920294@kmuh.org.tw; 2 Doctoral Degree Program in Toxicology, College of Pharmacy, Kaohsiung Medical University, Kaohsiung 807, Taiwan; 3 Multidisciplinary Swallowing Center, Kaohsiung Municipal Siaogang Hospital, Kaohsiung Medical University, Kaohsiung 812, Taiwan; 1116080@kmuh.org.tw; 4 Department of Pediatrics, Kaohsiung Municipal Siaogang Hospital, Kaohsiung Medical University, Kaohsiung 812, Taiwan; 940269@kmuh.org.tw; 5 Research Center for Precision Environmental Medicine, Kaohsiung Medical University, Kaohsiung 807, Taiwan; 6 Department of Pediatrics, Faculty of Medicine, College of Medicine, Kaohsiung Medical University, Kaohsiung 807, Taiwan; 7 Department of Pediatrics, Kaohsiung Medical University Hospital, Kaohsiung Medical University, Kaohsiung 807, Taiwan; 8 Department of Dentistry, Kaohsiung Medical University Hospital, Kaohsiung Medical University, Kaohsiung 807, Taiwan; 870127@kmuh.org.tw; 9 Department of Nursing, Fooyin University, Kaohsiung 831301, Taiwan

**Keywords:** cheek bulging, frailty, grip strength, oral frailty, swallowing dysfunction

## Abstract

**Background**: Swallowing dysfunction is a prevalent but often overlooked problem in long-term care facilities. This study investigated the relationships between nutritional deficits, frailty, oral health, and swallowing dysfunction. Additionally, we explored the associations between oral muscle weakness, frailty markers, and swallowing dysfunction. **Methods**: We recruited 373 participants from seven long-term care facilities across Taiwan. Swallowing function, frailty, nutrition status, and oral health were assessed by research staff. Oral muscle function was evaluated through tongue strength measurements, cheek bulging function tests, the Repetitive Saliva Swallowing Test (RSST), and a diadochokinetic task. Frailty was assessed through grip strength as well as mid-upper arm and calf circumference measurements. **Results**: The Functional Oral Intake Scale revealed that 97 participants (26%) had swallowing dysfunction. Participants with swallowing dysfunction had poorer nutritional status, higher frailty levels, and worsened oral health. Frailty was the factor most strongly associated with swallowing dysfunction. Participants with swallowing dysfunction also exhibited lower tongue pressure, decreased cheek-bulging ability, fewer repetitions in the diadochokinetic task, lower scores on the RSST, lower calf circumferences, and lower grip strength. Logistic regression demonstrated that cheek bulging was most strongly associated with swallowing dysfunction. Furthermore, lower grip strength was significantly associated with swallowing dysfunction. **Conclusions**: Frailty was most strongly associated with swallowing dysfunction, followed by poorer nutritional status and worsened oral health. These factors should be thoroughly assessed in long-term care residents. Participants with swallowing dysfunction also experienced oral muscle weakness, particularly in cheek bulging. Grip strength, which represents frailty, was directly associated with swallowing dysfunction and could serve as a crucial indicator of swallowing dysfunction.

## 1. Introduction

Swallowing dysfunction is subjectively reported by patients and objectively diagnosed by clinicians as an impairment in swallowing that results in an abnormal delay in the transit of a liquid or solid bolus from the oral cavity to the stomach [1]. It occurs in patients with cardiovascular disorder, Parkinson’s disorder, head injury, supranuclear palsy, head or neck postoperative period, etc. [1]. The prevalence of swallowing dysfunction among older adults ranges from 11% to 80%, depending on the selection criteria for study groups [2]. This prevalence can also vary among older adults in community settings, admitted to hospital, and in institutional care facilities [3]. Furthermore, swallowing dysfunction in older adults is associated with poorer nutrition, poor oral health, and frailty, impairing their daily life functioning [3]. The prevalence of swallowing dysfunction is high among older adults in long-term care facilities, especially those with comorbid degenerative or cerebrovascular diseases [4]. However, the prevalence of swallowing dysfunction and its association with frailty and oral health have not been well evaluated in long-term care centers in Taiwan. Accordingly, assessing swallowing dysfunction in these residents is crucial for identifying and addressing their specific treatment needs.

### 1.1. Swallowing Dysfunction in Residents in Long-Term Care Facilities

Previous reviews have indicated a lower quality of life among institutionalized older adults [5]. Long-term care center is one institutional case providing 24 h supervision and medical care and social interaction. Specifically, 43% of older patients in long-term care facilities reportedly experience oropharyngeal dysphagia and aspiration [4]. Swallowing dysfunction can contribute to nutritional deficits, functional impairments, and social and emotional challenges in older patients [6]. However, restrictions on staffing and insufficient physical and social activities cause challenges in detecting and addressing swallowing dysfunction in long-term care residents [7,8]. Furthermore, invasive assessments, such as Barium swallow or videofluoroscopic study, are impractical for long-term care residents with compromised health. Hence, implementing a practical method for evaluating swallowing dysfunction in these facilities to reduce complications, morbidity, disability, and mortality is essential [6].

### 1.2. Deficits in Nutrition in Older Patients in Long-Term Care Facilities

The prevalence of malnutrition has been reported to range from 6.5% to 48.3% in hospitals and 3.2% to 61.0% in other institutional care facilities [9], which is higher than that among noninstitutionalized older adults [10]. Factors such as poor oral intake [9], depression, cognitive impairment [11], and limited staff resources contribute to the risk of malnutrition. Older individuals with swallowing dysfunction are at an even greater risk of malnutrition owing to their reduced ability to consume adequate nutrients [12]. Furthermore, both swallowing dysfunction and poor oral health are strong predictors of malnutrition in older residents of care facilities [13]. If left untreated, malnutrition in older adults with swallowing dysfunction can lead to poor outcomes and increased healthcare costs [14]. Accordingly, assessing the nutritional statuses of older residents with swallowing dysfunction in long-term care facilities is essential.

### 1.3. Oral Health and Swallowing Dysfunction

Swallowing dysfunction is associated with oral health, with poor oral health contributing to swallowing difficulties [15]. Problems with teeth, gums, or oral infections can substantially impair the ability to chew and prepare food for swallowing [16]. The association between swallowing dysfunction and oral health is likely bidirectional and complex [17]. Both swallowing dysfunction and poor oral health have been shown to increase mortality rates in older adults [18]. Therefore, addressing oral health is essential in the management and treatment of swallowing dysfunction [16].

### 1.4. Frailty and Swallowing Dysfunction

Frailty is a syndrome characterized by decreased physiological reserve and resilience to stressors, leading to increased vulnerability to adverse health outcomes [19]. It is a critical concern in institutionalized older adults because it is strongly linked to negative outcomes, including disability, dependency, and comorbidities [20]. Frailty is highly prevalent, affecting approximately 52.3% of older adults in institutionalized care [20]. Frailty and swallowing dysfunction (dysphagia) are closely associated, particularly in older adults [21]. Frail individuals are at greater risk of dysphagia owing to declining muscle mass [22], and dysphagia can further contribute to the progression of frailty [23]. Frail older patients with oropharyngeal dysphagia often experience poor outcomes and higher mortality rates [24]. These findings indicate the importance of early identification and management of both frailty and dysphagia to improve health outcomes in older adults.

### 1.5. Oral Muscle Weakness and Swallowing Dysfunction

The oral phase of swallowing involves the coordinated action of muscles in the lips, tongue, cheeks, and soft palate, which manipulate food to form a bolus and propel it toward the pharynx to initiate swallowing. Weakness in these muscles can impair control over these processes, making it difficult to form and initiate swallowing [25,26]. In particular, weakened tongue muscles can delay the swallowing reflex and contribute to dysphagia owing to their critical role in bolus propulsion during the oral phase [27]. In addition, decreased tongue pressure has been linked to frailty [28] and malnutrition [29]. Cheek bulging function has also been associated with dysphagia [25]. Hence, assessing oral muscle function, including tongue pressure and cheek bulging, is essential for evaluating the risk of swallowing dysfunction and its association with frailty and malnutrition.

### 1.6. Grip Strength in Frailty and Swallowing Dysfunction

Grip strength is a well-established indicator of overall strength and a critical component of physical frailty [30]. It is widely recognized and validated as a measure of frailty, particularly in older adults, as one of the key criteria in the frailty phenotype [19]. Low grip strength is strongly associated with adverse health outcomes, including increased risk of inflammation, poor nutritional status, disuse, and depression, all of which can contribute to increased mortality risk [31]. In addition, grip strength has been associated with the risk of swallowing dysfunction because it reflects whole-body muscle strength [32] and is associated with tongue pressure [33]. However, whether grip strength is directly associated with swallowing dysfunction or indirectly through oral muscle weakness remains unclear.

The objective of this study was to investigate the prevalence of swallowing dysfunction among long-term care facility residents and its association with nutritional status, frailty, and oral health. We also assessed oral motor function and grip strength to explore their association with swallowing dysfunction.

## 2. Methods

This study was supported by the National Health Research Institutes and used purposive sampling to recruit participants from seven long-term care facilities across Taiwan. The institutions involved were Baihe Veterans Home, Zhongzhang Veterans Home, Penghu Home for the Elderly, Jiali Veterans Home, Tianzhong Veterans Home, Taitung Canaan Nursing Home, and Qijin Hospital Affiliated Long-term Care Facility. These long-term care centers are funded by the government or foundations, have capacities larger than 100 residences, and agreed to accept assessment and further follow-up for their residences. A total of 373 participants were recruited. Informed consent was obtained from all participants prior to evaluation, and the study was approved by the Institutional Review Board of Kaohsiung Medical University Hospital (KMUHIRB-SV(I)-20210041, approved on 9 July 2021).

### 2.1. Participants

All participants recruited for this study were confirmed by the research nurse to have tongue mobility, clear consciousness, the ability to express their thoughts and feelings clearly in Mandarin or Taiwanese, and the capacity to follow instructions to complete various clinical assessments. Individuals with mental illness, unstable medical conditions, or unclear consciousness were excluded. An unstable medical condition indicated an unpredictable, rapidly changing, or not well-controlled condition which posed an immediate or significant risk, such as unstable angina, delirium, or pneumonia. A total of 373 participants completed the questionnaire and underwent oral examinations between December 2021 and September 2023.

### 2.2. Measures

The demographic variables and clinical characteristics recorded during the initial evaluation included sex, age, education level, and the presence of tubes. In addition, mid-upper-arm circumference, calf circumference, height, and weight were documented as relevant associated variables.

Functional Oral Intake Scale: The Functional Oral Intake Scale (FOIS), a rating system developed by the Florida Health Science Center in 2005, assesses oral feeding function in stroke patients with dysphagia [34]. This 7-point ordinal scale describes typical patterns of functional oral intake [35], ranging from nonoral feeding (Levels 1 to 3) to various levels of oral feeding without supplementation (Levels 4 to 7) while considering dietary modifications and compensatory strategies used by patients. The agreement between paired judges of FOIS ranged from 85% to 95% [34]. In the present study, a score below 6 on the FOIS, out of a maximum of 7, was considered to indicate swallowing dysfunction [35].

Mini Nutritional Assessment-Short Form: The Mini Nutritional Assessment-Short Form (MNA-SF) is a validated tool commonly used in clinical and research settings to rapidly assess the nutritional statuses of older patients and identify those at risk of malnutrition. It comprises six items, such as “Has the patient experienced psychological stress or acute illness in the past 3 months?” Each item is evaluated on the basis of specific criteria and responses from the patient or caregiver. MNA-SF scores range from 0 to 14, with higher scores indicating better nutritional status and a lower risk of malnutrition. The Cronbach’s alpha coefficient for the MNA-SF has typically been reported to be approximately 0.80 or higher, reflecting strong internal consistency and reliability [36].

Study of Osteoporotic Fractures method for frailty assessment. The Study of Osteoporotic Fractures (SOF) method is widely used to assess frailty in older adults and helps to identify individuals at risk of adverse health outcomes. The SOF index is determined by the presence of two or more of the following three components [37]: (1) weight loss of 5% or more between the baseline and a follow-up examination, (2) inability to perform five chair rises without using the arms, and (3) answering “no” to the question “Do you feel full of energy?” on the Geriatric Depression Scale. Men with none of these components are classified as robust, whereas those with one component are considered in an intermediate stage of frailty [38].

Oral Health Assessment Tool: The Oral Health Assessment Tool (OHAT) was developed by Chalmers et al. [39] and evaluates oral health across eight categories: lips, tongue, gums and tissues, saliva, natural teeth, dentures, oral cleanliness, and dental pain. Each category is rated on a scale from 0 to 2, where 0 indicates a healthy situation, 1 indicates changes, and 2 indicates an unhealthy situation. The total score across the eight categories ranges from 0 to 16, with lower scores indicating better oral health. A score of 1 or higher suggests that the individual should see an oral health-care professional [39]. The Cronbach’s α value for OHAT was reported to be 0.60.

Tongue pressure: In this study, IOPI Pro (Iowa Oral Performance Instrument Medical, WA, USA), an effective and objective tool for assessing tongue and oral muscle strength by utilizing an air-filled tongue bulb, was used to assess tongue pressure. The IOPI Pro quantifies the maximum pressure exerted by the tongue against the palate, displaying the peak pressure in kilopascals (kPa) on an easy-to-read digital screen [40]. This device primarily measures oral function and tongue muscle strength. During the test, the measuring balloon was positioned in the center of the tongue and secured by the researcher. The participant was instructed to press their tongue firmly against the hard palate for 5 s. The test was repeated three times, with 30 s of rest between trials, and the highest value recorded was used to represent the participant’s maximal tongue pressure. A tongue pressure value below 30 kPa, the median, was considered indicative of reduced tongue strength [40,41].

Grip strength: The grip strength of the participant’s dominant hand was measured using an electronic dynamometer (CAMRY EH101; Zhongshan Camry Electronic Co., Ltd., Zhongshan, China), a high-precision strain gauge sensor, with readings up to 90 kg (198 lbs) and with a division of 0.1 kg (0.2 lbs). It featured an adjustable handle to accommodate various hand sizes. The participant was instructed to keep their shoulders relaxed, with the elbow flexed at 90° and the forearm in a neutral position. Maximal grip strength was measured, and the highest value from three trials, each separated by a 1 min interval, was used for analysis.

Cheek bulging: We assessed the strength, symmetry, and control of the buccinator muscles by asking the participant to puff out their cheeks. A score of 3 was assigned if the patient could puff out both cheeks normally, 2 if they could do so only partially (such as weakly or asymmetrically), and 1 if they were unable to do it.

Repetitive Saliva Swallowing Test: The Repetitive Saliva Swallowing Test (RSST), introduced in Japan by [42], is a screening test where the patient is asked to swallow saliva as many times as possible within 30 s. The RSST was assessed by a research nurse, a speech therapist, and a nutritionist, all of whom had undergone training that included theoretical instruction, observation of demonstrations, and supervised practice. Fewer than two swallows was considered abnormal. During the test, the rater palpated the patient’s laryngeal prominence and hyoid bone with two fingers to count the number of swallows.

Diadochokinetic test: The diadochokinetic (DDK) test evaluates articulatory and rhythmic speech impairments by having a participant rhythmically repeat a consonant–vowel sequence (e.g., PA-TA-KA) that involves different places of articulation: bilabial, alveolar, and velar. The assessment typically includes three items, each corresponding to one syllable: “pa”, “ta”, and “ka”. For example, the participant is instructed to say “pa” as many times as possible within 5 s. The number of accurate repetitions within that time frame is recorded for each syllable, with scores ranging from 0 to a practical maximum of approximately 30 repetitions in 5 s.

### 2.3. Statistical Analysis

Data analysis was conducted using SPSS v26. The statistical data included demographic characteristics of the participants, such as sample size, mean age, standard deviation, and variable distributions. In addition, the percentage of participants at each level of swallowing dysfunction, as assessed by the FOIS, was calculated for all participants, for those aged 80 years old or more, and for others. An independent *t*-test was used to assess differences in body mass index, nutritional status, frailty, grip strength, mid-upper-arm circumference, calf circumference, DDK score, tongue pressure, cheek bulging, RSST score, and oral health between participants with and without swallowing dysfunction. Logistic regression was then performed to identify the factors most associated with swallowing dysfunction, focusing on nutritional status, frailty, and oral health. Pearson’s correlation was used to assess the relationships between grip strength, DDK score, tongue pressure, cheek bulging, and RSST score. Logistic regression was also employed to evaluate the association between grip strength and swallowing dysfunction; oral muscle weakness was controlled for in this evaluation. A *p* value < 0.05 was considered significant in all analyses.

## 3. Results

A total of 373 residents completed the study evaluation, of whom 72.4% (270 individuals) were men; the average age of the residents was 82.83 years. Moreover, 43.7% of the participants (*n* = 163) had high school or vocational school as their highest educational level (Table 1). On the basis of the FOIS, 97 participants (26%) were identified as having swallowing dysfunction, of whom 7 (7.2%) required oral intake supplemented or dependent on a feeding tube, with the remaining participants requiring special food preparation. There was no difference in the distribution between those aged 80 or more and others.

### 3.1. Nutrition Deficit, Frailty, and Oral Health

The independent *t*-test revealed that participants with swallowing dysfunction had significantly lower body mass indexes (t = −2.78, *p* = 0.01), poorer nutritional status (t = −2.26, *p* = 0.02), reduced grip strength (t = −4.48, *p* < 0.001), and poorer oral health (t = −2.02, *p* = 0.04), while exhibiting higher levels of frailty (t = 5.19, *p* < 0.001; Table 2). Our logistic regression analysis, conducted to assess the association between nutritional status, frailty, oral health, and swallowing dysfunction, indicated that frailty was the strongest associated factor, followed by impaired oral health and nutritional deficits (Model 1, Table 3). Participants with greater frailty [odds ratio (OR) = 1.87, 95% confidence interval (CI) = 1.37–2.54], poorer oral health (OR = 1.18, 95% CI = 1.05–1.33), and lower nutritional status (OR = 0.93, 95% CI = 0.87–0.99) were more likely to experience swallowing dysfunction.

### 3.2. Oral Muscle Weakness and Grip Strength

The independent *t*-test demonstrated that participants with swallowing dysfunction had significantly lower grip strength (t = −4.48, *p* < 0.001); fewer repetitions of “pa” (t = −2.93, *p* = 0.04), “ta” (t = −3.31, *p* = 0.01), and “ka” (t = −3.57, *p* < 0.001); lower tongue pressure (t = −3.61, *p* < 0.001); reduced ability to puff out their cheeks (t = −5.40, *p* < 0.001); and fewer repetitions in the RSST (t = −2.23, *p* = 0.03). Grip strength was significantly correlated with tongue pressure (r = 0.37, *p* < 0.001); ability to puff out the cheeks (r = 0.23, *p* < 0.001); and the number of repetitions of “pa” (r = 0.35, *p* < 0.001), “ta” (r = 0.35, *p* < 0.001), and “ka” (r = 0.29, *p* < 0.001).

Our forward logistic regression (Model 2, Table 3) indicated that the ability to puff out the cheeks was the strongest predictor of swallowing dysfunction (OR = 0.53, 95% CI: 0.37–0.75), followed by the number of repetitions of “ka” (OR = 0.74, 95% CI: 0.58–0.93). This suggests that cheek bulging is the most strongly associated factor in oral muscle weakness related to swallowing dysfunction. Participants with reduced ability to puff out their cheeks and pronounce “ka” were more likely to have swallowing dysfunction. After controlling for these factors, we observed that grip strength remained significantly associated with swallowing dysfunction (OR = 0.94, 95% CI: 0.91–0.98; Model 3, Table 3).

## 4. Discussion

### 4.1. Swallowing Dysfunction

This study demonstrated that 26% of residents in long-term care facilities experienced swallowing dysfunction, as assessed by the FOIS. This result is consistent with previous reports of swallowing issues among hospitalized elderly populations [40]. Of those with swallowing dysfunction, 7.2% required tube supplements, and the remaining required specially prepared food. These findings indicate that swallowing dysfunction is a prevalent problem in long-term care facilities in Taiwan. Therefore, routinely assessing swallowing function using practical, standardized tools such as the FOIS in these settings is essential.

### 4.2. Frailty, Nutrition Deficit, and Oral Health in Swallowing Dysfunction

Previous studies have demonstrated that frailty, nutritional deficits, and poor oral health are associated with an increased risk of swallowing dysfunction in hospitalized elderly patients [43], and the present study revealed similar findings. Numerous studies have highlighted the complex association between frailty, nutritional deficits, oral health, and swallowing dysfunction [15,17,23]. Medical comorbidities, such as stroke-related neuromuscular impairments, may contribute to these four dimensions of impairment and explain their interconnection. Additionally, swallowing dysfunction can limit food intake, exacerbating frailty and nutritional deficits. The interplay among frailty, nutritional deficits, oral health, and swallowing dysfunction suggests that these factors not only influence each other, but may also be mutually reinforcing. Environmental and social factors, such as limited resources for intervention and inadequate communication or motivation among staff in long-term care facilities, can further compound these problems. The cumulative effect of these challenges contributes to increased mortality and morbidity [8,18]. Considering the strong association between frailty, nutritional deficits, oral health, and swallowing dysfunction, thoroughly assessing these factors in residents with swallowing dysfunction in long-term care facilities is vital.

### 4.3. Frailty and Oral Muscular Function in Swallowing Dysfunction

We demonstrated that long-term care residents exhibited lower tongue pressure, reduced salivary swallowing capacity, impaired diadochokinetic task performance, and diminished cheek-bulging ability. These findings suggest that they have compromised oral muscle function, which contributes to their swallowing dysfunction. The results also highlight the importance of proper coordination between the tongue, cheeks, salivation, and pronunciation for maintaining swallowing function. Furthermore, our findings reveal that cheek bulging is the oral muscle factor most strongly associated with swallowing dysfunction, followed by pronunciation difficulties with the syllable “Ka”. Cheek muscles play a crucial role in pushing the food bolus toward the throat and coordinating with other muscles to complete the swallowing process. Weakness in the cheek muscles can lead to improper bolus formation, difficulty in swallowing, or even obstruction during swallowing. This essential role of cheek muscles in the swallowing process accounts for their strong association with swallowing dysfunction observed in this study.

Frailty was the strongest factor associated with swallowing dysfunction in this analysis, reinforcing the close connection between frailty and swallowing dysfunction, as documented in previous studies [23,28,44]. A previous systematic review indicated that deterioration in oral health, decline in oral motor skills, impaired swallowing function, and oral pain could contribute to frailty, forming a subtype known as oral frailty [45]. Additionally, studies have demonstrated an association between tongue pressure and grip strength, demonstrating the role of oral function in frailty [33]. Grip strength has been reported to correlate with tongue pressure [46] and to be an indicator of swallowing dysfunction [47]. Our results similarly demonstrate that grip strength, an indicator of frailty, was associated with tongue pressure; cheek-bulging ability; and number of repetitions for “pa”, “ta”, and “ka”. The sarcopenia leads to overall muscle weakness and underlines the associations mentioned above. On the other hand, the low tongue pressure or cheek bulging contributes to dysphagia, which can lead to malnutrition and, thus, contribute to frailty. These findings indicate a close relationship between frailty and oral muscle function, supporting the concept of oral frailty [48], which refers to dysphagia associated with general sarcopenia and a decline in oropharyngeal muscle mass.

Our results also demonstrate that grip strength was associated with swallowing dysfunction, even when oral muscle weakness was controlled for. This finding indicates a direct effect of grip strength on swallowing dysfunction, which is not mediated by oral muscular weakness. This finding confirms the crucial role of sarcopenia in swallowing dysfunction, supporting the concept of oral frailty. Consequently, grip strength could be a crucial clinical indicator for swallowing dysfunction, as previously reported [47].

### 4.4. Clinical Implications

Our results reveal a close association between frailty, nutritional deficits, oral health, and swallowing dysfunction, all of which share underlying etiologies such as aging, comorbidities, sarcopenia, and psychosocial factors. This suggests that these four areas of impairment should be routinely assessed in elderly individuals residing in long-term care facilities, particularly in settings with limited staff, resources, and medical interventions. This study also presented a practical way to assess early sign of swallowing dysfunction in long-term care centers with limited resources. Given their bidirectional interactions, addressing one of these dimensions may positively influence the others. Thus, multidisciplinary collaborative interventions to improve frailty, such as resistance training or balance exercise; to optimize one’s nutrition status, such as a protein-rich diet and vitamin support; and to maintain oral health, such as oral hygiene and dental care, should be provided as early as possible to prevent oral dysfunction. In line with the concept of oral frailty, grip strength should be assessed to evaluate the role of frailty in swallowing dysfunction, particularly for the early detection of pre-dysphagia. Impairments in cheek bulging and tongue pressure, which may be part of general frailty, should also be assessed. Early rehabilitation or practical training interventions, such as oral exercise, targeting these oral muscle functions could help to preserve swallowing ability and prevent further decline.

### 4.5. Limitations

This study has several limitations. First, because swallowing dysfunction and its associated factors were measured simultaneously, a causal relationship could not be confirmed in this presenting study. A prospective study design should be considered in the future to reveal the temporal relationship between swallowing dysfunction and these associative factors. Second, the participants of the study were not recruited based on random or stratified sampling. The diversity in geographical distribution, local healthcare infrastructure, diet, or cultural practices could affect the generalizability of this study to other populations. Several potential confounding factors, such as medication use, laboratory data, and general health status, were not available in this study. They should be controlled in future studies. Finally, the findings were based on surveys conducted in specific regions of Taiwan, which may limit their generalizability to other populations.

## 5. Conclusions

A total of 26% of residents in long-term care facilities were found to experience swallowing dysfunction. These participants were more likely to experience frailty, nutritional deficits, and poor oral health. Considering these associations, swallowing dysfunction and its related impairments should be routinely assessed in long-term care residents. Notably, frailty was the most significant factor linked to swallowing dysfunction, and participants with this condition exhibited oral muscle weakness and reduced grip strength. Even when oral muscle weakness was controlled for, grip strength was still directly associated with swallowing dysfunction. These findings indicate that grip strength could be a crucial indicator of swallowing dysfunction and should be regularly evaluated in this population.

## Figures and Tables

**Table 1:** Summary of demographic characteristics and the level of swallowing dysfunction of long-term care facility residents (*n* = 373).

Category			
Number of People	*n* = 373		
Average Age	82.83 years old		
Age Standard Deviation	10.98 years		
Age Range (Min–Max)	39–104 years old		
Gender Ratio (M/F)	2.62		
Education Level	*n* (%)		
Illiterate/Literate but not in school	70 (18.8)		
Elementary/Junior High School	135 (36.2)		
High School/College or above	163 (2.32)		
Missing Values	5 (1.3)		
Swallowing dysfunction (FOIS)	All: *n* (%)	<80 y/o: *n* (%)	≥80 y/o: *n* (%)
No oral intake	0 (0)	0 (0)	0 (0)
Tube dependent with minimal/inconsistent oral intake	3 (0.8)	2 (1.2)	1(0.4)
Tube supplements with consistent oral intake	4 (1.1)	4 (3)	0 (0)
Total oral intake of a single consistency	44 (11.8)	12 (9)	32 (13.3)
Total oral intake of multiple consistencies requiring special preparation	46 (12.3)	16 (12)	30 (12.5)
Total oral intake with no special preparation, but must avoid specific foods or liquid items	65 (17.4)	24 (18)	41 (17.1)
Total oral intake with no restrictions	211 (56.6)	75 (56.4)	136 (56.7)

**Table 2:** The nutrition status, frailty, oral health, and oral muscle function of participants with swallowing dysfunction in Functional Oral Intake Scale (FOIS).

Variables	*FOIS*		Independent t
	Abnormal *n* = 97 *n* (%) Mean ± SD (Missing)	Normal *n* = 276 *n* (%) Mean ± SD (Missing)	
Gender			
Male	198 (77.3%)	72 (26.7%)	0.222
Female	78 (75.7%)	25 (24.3%)	
Age	83.46 ± 11.55	82.60 ± 10.78	0.659
BMI	22.40 ± 3.98 (1)	23.65 ± 3.75 (8)	−2.776 **
Nutrition status	9.68 ± 3.58	10.72 ± 4.0 (1)	−2.262 *
Oral Health	4.59 ± 2.59	3.74 ± 2.40	2.932 **
Frailty	1.30 ± 1.05 (4)	0.70 ± 0.79 (48)	5.185 ***
Grip strength (kg)	13.19 ± 8.15 (5)	17.94 ± 8.99 (3)	−4.48 ***
Mid-upper-arm circumference	25.07 ± 4.82	25.81 ± 4.50	−1.35
Calf circumference	29.41 ± 6.02	31.39 ± 4.93	−3.19 **
Tongue pressure	18.17 ± 13.95 (6)	24.93 ± 15.87 (12)	−3.609 ***
RSST	3.23 ± 2.87 (10)	4.06 ± 3.06 (9)	−2.23 *
PA	2.25 ± 1.21 (10)	2.72 ± 1.32 (10)	−2.928 **
TA	2.17 ± 1.28 (13)	2.70 ± 1.30 (13)	−3.314 **
KA	2.13 ± 1.18 (12)	2.68 ± 1.25 (14)	−3.567 ***
Cheek bulging	2.11 ± 0.86 (1)	2.57 ± 0.65 (6)	−5.40 ***

*: *p* < 0.05; **: *p* < 0.01; ***: *p* < 0.001. Nutritional status: the score of Mini Nutritional Assessment-Short Form. Oral Health: the score of Oral Health Assessment Tool. Frailty: the score of Study of Osteoporotic Fractures Activity of Daily Living. RSST: repetitive saliva-swallowing test. PA/TA/KA: number of pronunciations within five s.

**Table 3:** The logistic regression for the association between associated variables and swallowing dysfunction in the Functional Oral Intake Scale (FOIS).

FOIS Abnormal	Wald χ^2^	Odds Ratio (95% CI)
Model 1 (Swallowing dysfunction regressed with frailty, oral health, and nutritional status)		
Gender	0.60	0.79 (0.43–1.45)
Age	0.28	0.99 (0.7–1.02)
Frailty	15.82 ***	1.87 (1.37–2.54)
Oral Health	7.45 **	1.18 (1.05–1.33)
Nutrition status	4.48 *	0.93 (0.87–0.99)
Model 2 (Swallowing dysfunction regressed with factors of oral muscle weakness)		
Gender	0.19	1.15 (0.62–2.11)
Age	0.50	1.01 (0.99–1.03)
Cheek bulging	12.34 ***	0.53 (0.37–0.75)
Ka	6.63 *	0.74 (0.58–0.93)
Model 3 (Swallowing dysfunction regressed with grip strength in control of oral muscle weakness)		
Gender	2.38	1.67 (0.87–3.21)
Age	0.31	1.01 (0.98–1.03)
Grip Strength	9.43 **	0.94 (0.91–0.98)
Cheek bulging	7.99 **	0.58 (0.40–0.85)
Ka	3.08	0.80 (0.63–1.03)

*: *p* < 0.05; **: *p* < 0.01; ***: *p* < 0.001. Nutritional status: the score of Mini Nutritional Assessment-Short Form. Oral health: the score of Oral Health Assessment Tool. Frailty: the score of Study of Osteoporotic Fractures Activity of Daily Living. KA: number of pronunciations within five s.

## Data Availability

The data presented in this study are available upon request from the corresponding author due to restrictions imposed by IRB regulations.
